# Evaluation of Rice Degree of Milling Based on Bayesian Optimization and Multi-Scale Residual Model

**DOI:** 10.3390/foods11223720

**Published:** 2022-11-19

**Authors:** Weidong Chen, Wanyu Li, Ying Wang

**Affiliations:** 1 College of Information Science and Engineering, Henan University of Technology, Zhengzhou 450001, China; 2 National Engineering Research Center for Grain Storage and Logistics (Wheat), Zhengzhou 450001, China

**Keywords:** degree of milling, multi-scale information fusion, residual network model, Bayesian optimization algorithm

## Abstract

Traditional machine learning-based methods for the detection of rice degree of milling (DOM) that are not comprehensive in feature extraction and have low recognition rates fail to meet the demand for fast, non-destructive, and accurate detection. This paper presents a digital image processing technology combined with deep learning to implement the classification of DOM of rice. An improved multi-scale information fusion model of the InceptionResNet–Bayesian optimization algorithm (IRBOA) was constructed based on the Inception-v3 structure and residual network (ResNet) model. It enables to automatically extract more comprehensive features of rice and determine the DOM of rice. Additionally, the important hyperparameters in the model were tuned by the BOA to optimize the recognition rate of rice DOM. The results show the hyperparameters optimized using the BOA are those that would not be chosen in manual tuning. The classification precision of the IRBOA model reached 99.22%, 94.92%, and 96.55% for well-milled, reasonably well-milled, and substandard rice, respectively, with an average accuracy of no less than 96.90%. This model improved 7.41% over the traditional machine learning model and at least 1.35% over the fashionable CNN model with strong generalization performance. This method effectively completes rapid, non-destructive, and accurate intelligent detection of rice DOM, which can supply a reliable and accurate technical mean for rice processing enterprises to guide the rice processing process.

## 1. Introduction

Paddy is a major grain in the world. As the worldwide population grows, the requirement for rice is expected to rise by 30% in 2050 [1]. Therefore, the processing and production of rice have a vital role. At present, there are prominent problems in the rice market, such as the one-sided pursuit of appearance quality (fine, white, and nice taste), backward control means of the DOM, and nutrient loss caused by over-processing, which threaten food security [2]. Thus, an efficient and rapid method of estimating the DOM of rice can instruct enterprises to adjust the parameters in the rice milling process in real-time. Additionally, enterprises can perform such approaches to moderately process rice and achieve efficient rice loss reduction through technological innovation. It has essential significance for guiding paddy processing, rice storage, distribution, and trade.

According to the regulations of the Chinese National Standard of “Milled rice (GB/T 1354-2018) [3]”, rice DOM refers to the degree of germ remaining and the residual bran layer on the surface and back grooves of a rice grain after processing, which is divided into three levels: well-milled, reasonably well-milled, and substandard. Well-milled, reasonably well-milled, and substandard rice represent rice with skin retention less than 2%, between 2% and 7%, and more than 7%, respectively. The skin retention of rice is defined as the sum of the residual skin and rice embryo projection area as a percentage of the projection area of the sample. In rice processing enterprises, detecting the DOM of rice is still at the stage of human eye inspection or staining method to auxiliary implementation. These approaches have the disadvantages of strong professionalism, being time-consuming and labor-intensive, poor repeatability, etc. Foreign researchers found that rice DOM is closely related to its chemical composition content [4]. They extracted the lipid content of the milled rice surface by chemical extraction to quantify the DOM of rice [5]. However, this method cannot meet the requirements of modern rice DOM for rapid, non-destructive, efficient, and objective detection.

Machine vision technology provides the advantages of high efficiency, fast speed, and accurate detection, which is currently a research hot spot in the field of crop detection [6,7,8]. Xu et al. [9] and Wood et al. [10] detected the DOM of rice by digital image processing technology combined with the staining method, but the staining process was cumbersome and destructive. Zhang et al. [11] obtained the rice DOM by the bran degree of RGB images of rice. Wan and Long [12] and Wan et al. [13] proposed detection methods based on gray-gradient co-occurrence matrix and color features incorporated with machine learning, respectively, and the corresponding discrimination accuracy reached 94% and 92.17%. Fang et al. [14] used grayscale values of rice to measure DOM. Zareiforoush et al. [15] adopted the fuzzy logic reasoning method to realize the recognition of five rice milling grades, and the overall confidence reached 89.80%. Hortinela et al. [16] used the support vector machine to classify milled rice with an adaptive enhancement algorithm, and the average accuracy was 86.67%. Although the above methods achieved positive detection results, they all need to design and extract features manually, and there is the problem that incomplete feature extraction leads to low accuracy.

In recent years, CNN has achieved remarkable achievements in face recognition [17], handwritten digit recognition [18], pedestrian detection [19], and other fields, bringing new opportunities for the development of rice DOM detection technology. In terms of DOM detection of rice, Qi et al. [20] combined the hypercolumn technology, max-relevance and min-redundancy feature selection algorithm, extreme learning machine technique, and improved VGG16 to identify rice DOM with an overall accuracy of 97.32%. For the quality inspection of rice, Patel and Joshi [21] used the transfer learning-based VGG16 model for fine rice, broken rice, and variety determination. A four-layer CNN model to realize head and broken rice classification was adopted by Hong Son and Thai-Nghe [22]. Li and Li [23] improved Inception-v3 by introducing fine-grained classification to learn local features of rice and to identify the integrity of the rice germ. Li et al. [24] refined the Inception-v3 model to detect the integrity of the germ with the addition of mutual channel loss and mlpconv. Li et al. [25] identified rice germ integrity based on the EfficientNet-B3 model with the introduction of the double attention network (DAN).

To summarize, existing research on rice is mostly quality examination, while the determination of rice DOM has essential guidance for maintaining food nutrition and reducing food waste. The current research is unable to acquire the feature details of rice well, and there is still a lack of deep learning-based methods that can effectively and correctly identify the DOM of rice. Therefore, the main contributions of this study are as follows:

(1). Simple image preprocessing and single-grain rice segmentation methods are used to segment single-grain rice images from multiple-grain rice images. Then, they are fed into the improved IRBOA model for rice DOM classification.
(2). The Inception-v3 structure with ResNet34 are combined to fuse rice features at different scales and enrich the feature representation, thereby enabling the detection of rice DOM and enhancing the recognition accuracy of the model.
(3). We used BOA to search for the hyperparameters that lead to the optimal model performance in order to avoid the problem of manual setting of hyperparameters that fail to obtain the peak accuracy. The method can increase the discrimination rate of the model via upgrading the efficiency of manual search.

## 2. Materials and Methods

### 2.1. Experimental Materials and Image Acquisition

Standard samples of early indica rice DOM (SAC LS/T 15121-2020), including well-milled, reasonably well-milled and substandard, were selected from the Anhui grain and oil products quality supervision and testing station in Hefei, Anhui Province, China. A total of 50 g of each class of rice was used for sample preparation. Each five grams of rice was packed in a sealed bag as a group, and each type of rice was packed in 10 groups. Finally, there were 30 groups of three types of rice, marked with the corresponding serial numbers, and stored in a refrigerator at 0–5 °C to prevent the influence of sample deterioration on the inspection results.

**Figure 1:**
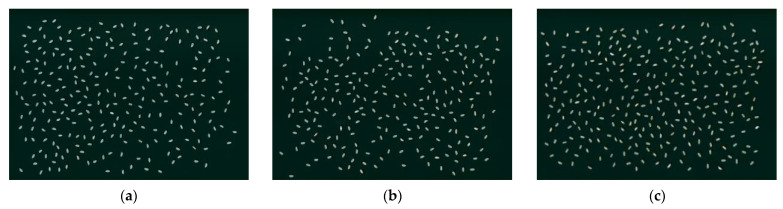
Images of the original multi-grain rice. (**a**) Well-milled. (**b**) Reasonably well-milled. (**c**) Substandard.

According to the requirements of rice image acquisition, a Phantom h9 flatbed scanner was used to acquire RGB images of rice in multiple mixed poses with the background of a black frosted Acrylic plate. The contrast ratio, brightness, resolution, and image size of the flatbed scanner were set to 65, 30, 600 dpi, and 5000 pixels × 7000 pixels, respectively. Image acquisition was carried out in units of five grams, and each group of rice was placed on the draft table of the scanner with the help of a separating sieve to avoid the adhesion of rice grains. Then, image scanning was performed. Next, the operation of random placement and scanning was executed again to fully utilize the sample and obtain two different images. Finally, the scanned rice was put into the corresponding sealed bag, and the other group of rice was repositioned on the scanner. The above steps were performed on 30 groups of samples of well-milled, reasonably well-milled, and substandard in turn. Finally, a total of 60 valid images were obtained, some of which are shown in Figure 1.

### 2.2. Image Preprocessing

The image quality of the original images of multi-grain rice is affected by noise due to the limitation of the shooting conditions. So, a series of preprocessing operations were taken for the images to selectively highlight effective features and eliminate irrelevant information in order to improve the image quality and increase the classification and recognition accuracy. Meanwhile, in this research, we performed image smoothing, binarization, and segmentation of single-grain rice on the original rice images before inputting the single-grain rice images into the CNN model.

#### 2.2.1. Image Smoothing and Binarization

We first converted each color image to grayscale using an image grayscale transform. Image smoothing was achieved by median filtering that can eliminate image noise while preserving image edge information before implementing image segmentation [26]. We used a fixed threshold to complete the image binarization operation, which avoided the situation of separating rice endosperm and bran by other methods. Finally, we performed a morphological opening and closing operation on the binarized image to smooth the image and fill the holes inside the target rice.

#### 2.2.2. Segmentation of Single-Grain Rice Images

**Figure 2:**
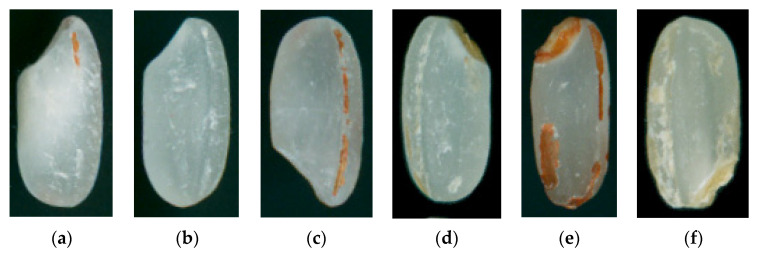
Single-grain rice images of three kinds of DOM. (**a**,**b**) Well-milled. (**c**,**d**) Reasonably well-milled. (**e**,**f**) Substandard.

The Canny algorithm of contour detection was used to detect the edge of each grain of rice. The minimum circumscribed rectangle of each rice was drawn, and its four vertex coordinates and rotation angle were gained. Next, the original rice image was rotated by the derived rotation angle. Finally, image segmentation of single-grain rice in a vertical state was realized by extending the coordinates of the rotated rectangle vertex to the surroundings by 5 pixels as the boundary. Figure 2 shows the sample data of three kinds of DOM rice after single-grain segmentation.

### 2.3. Data Augmentation

A dataset was established based on the segmented single-grain rice images, and 5800 valid images each of well-milled, reasonably well-milled, and substandard rice was obtained, for a total of 17,400 images. Each category of rice dataset was divided into a training set, validation set, and test set with a ratio of 6:2:2 for each category. That means obtaining 3480 images per class of rice for the training set and 1160 images for the validation and test sets, respectively. The training set is used for training the model, while the validation set is employed to optimize the model structure and hyperparameters, and the test set is only designed to test the performance of the model to enhance its generalization ability.

It is essential to enhance the training set data to reduce the incidence of overfitting when the data are limited. Firstly, each rice was cropped to an image of the same size (224 pixels × 224 pixels) by the center cropping for input into the CNN model. Secondly, 30% of the training data were randomly selected for horizontal and vertical flipping, respectively. Then, a random rotation was executed for each image with rotation angles ranging from 35° to 135°. Finally, the mean and standard deviation of the three color channels of all training set images were calculated and fed into the normalization function to realize the normalization of each image. The training set was expanded according to the above steps to derive sufficient data to train models.

### 2.4. Proposed Approach

CNN is one of the most popular deep learning models and is widely used in image classification tasks at present. It is not only able to extract features of target objects in images automatically and comprehensively but also possesses the characteristic of weight sharing, which reduces the training parameters of the network and makes the model simpler [27]. We constructed an IRBOA model which can fuse multi-scale information based on the integration of the Inception-v3 structure and ResNet model to classify rice from three kinds of DOM. The model used was as described below.

#### 2.4.1. Inception Structure

Inception structure is a significant breakthrough in the development history of CNN models. Its purpose is to execute multiple convolution operations or pooling operations on the input image in parallel and concatenate all the outputs to attain more comprehensive image features. This structure was first introduced by GoogLeNet and called Inception-v1 [28]. Subsequently, it was improved to the Inception-v2 structure by applying batch normalization (BN) [29] and convolutional decomposition. Then, it evolved into the Inception-v3 network by adding asymmetric convolution, auxiliary classifiers, etc. The architecture not only accelerates the computation but also improves the generalization ability of the model while eliminating the use of dropout in the batch normalization network [30]. Currently, the Inception structure has been developed to the Inception-v4 [31].

#### 2.4.2. ResNet Model

**Figure 3:**
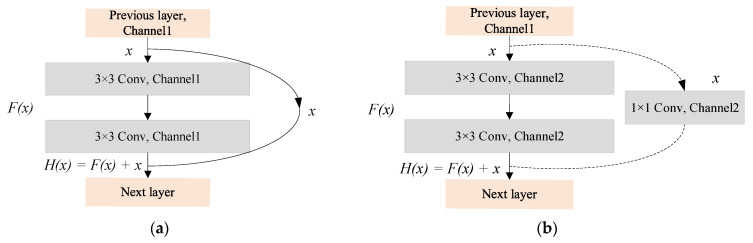
Residual structure. (**a**) Residual-A structure. (**b**) Residual-B structure.

ResNet, which emerged in 2015, marks a milestone in deep learning [32]. It adjusts the structure of the traditional CNN models, in which the most critical residual structure adds an identity mapping to the basic network unit [33]. The residual structures are shown in Figure 3. The original fitting target of the residual structure is H(x), and it becomes extremely difficult to learn H(x) with the gradual deepening of the network level. Thus, transforming the fitting target into the fitted residual function F(x) (F(x)=H(x)−x) through the residual structure and turning the output into a superposition of the fit, and the input will make the learning of the network relatively easy. The residual learning is adopted for each stacked layer in ResNet, and the residual learning formula is defined as:

(1) $$y=F\left(x,\left\{w_{i}\right\}\right)+x$$

where x and y are the input and output vectors of the residual structure of this layer, and $F\left(x,\left\{w_{i}\right\}\right)$ represents the residual mapping to be learned. For the example in Figure 3 that has two layers, $F=w_{2}ReLU\left(w_{1}x\right)$ in which ReLU denotes ReLU activation function. In addition, the dimensions of $F(x,\{w_{i}\})$ and x should be consistent. $w_{S}$, a square matrix, can be conducted through identity mapping to match the dimensions when the input or output dimension information needs to be changed, as shown in Figure 3b.

(2) $$y=F\left(x,w_{i}\right)+w_{s}x$$

#### 2.4.3. Custom Model

**Figure 4:**
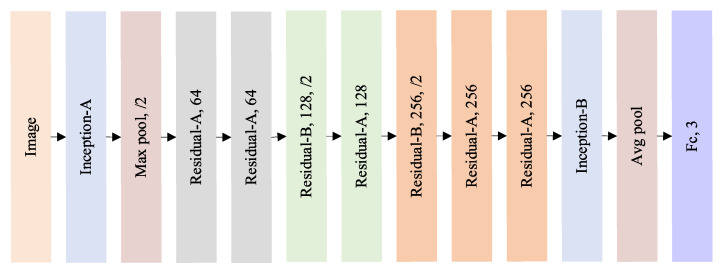
The architecture of the IRBOA model.

**Figure 5:**
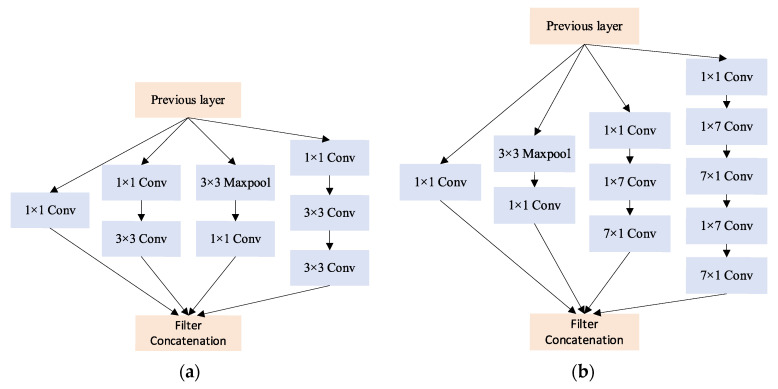
Inception-v3 structure. (**a**) Inception-A structure. (**b**) Inception-B structure.

The Inception-v3 structure offers the characteristics of fusing multi-scale features and accelerating network computation, while the residual structure in ResNet prevents gradient explosion, gradient disappearance, and network degradation when the number of network layers is deepened. Consequently, in this study, we integrated the Inception-v3 structure and residual module and established a multi-scale information fusion CNN model based on ResNet34 architecture, named InceptionResNet–BOA model, or IRBOA model for short. The model was adopted to enrich the rice feature information and promote the recognition effect. The structure of the IRBOA model is shown in Figure 4. The input of the model is a 224 × 224 × 3 color image, and the model architecture consists of an Inception-A structure as shown in Figure 5a, a maximum pooling layer, five Residual-A structures, two Residual-B structures, an Inception-B structure as shown in Figure 5b, and an average pooling layer. The input of the fully connected layer is the number of flattened characteristic maps of the average pooled layer. While the count of neurons of this layer is the amount of rice DOM types to classify rice DOM.

Table 1 displays the parameter settings for each layer of the IRBOA model. The Inception-A structure is a parallel combination of a series of 1 × 1 convolution layers, 3 × 3 convolution layers, and a 5 × 5 convolution layer replaced by two 3 × 3 convolution layers, with the number of convolution kernels from branch1 to branch4 being 8, 12, 24, 8, 12, 24, 24, respectively. The Residual-A structure contains two convolutional layers with 3 × 3 kernels and an identity mapping, and the number of convolutional kernels in Residual-A1 to A4 are 64, 128, 256, and 256, respectively. Residual-B structure matches the number of channels in the two pathways by 1 × 1 convolution at identity mappings based on the Residual-A structure, with 128 and 256 convolution kernels for Residual-B1 to B2. The Inception-B structure is combined by 1 × 1 convolution layers, asymmetric 1 × 7 convolution layers, and 7 × 1 convolution layers. The number of convolution kernels from branch1 to branch4 are 64, 128, 64, 64, 128, 192, 192, 192, 192, and 128, respectively.

### 2.5. Optimization Methods of the Model

#### 2.5.1. BOA

Determining how to select appropriate hyperparameters has become a key issue in image classification tasks in the circumstance that the performance of the model largely depends on the selection of hyperparameters. The method of manual optimization is difficult and time-consuming to find the optimal parameters. Recently, the widely used methods of automatic parameter tuning of machines include the grid search algorithm (GSA), the random search algorithm (RSA), and the BOA. The essence of the GSA is the enumeration method, which is costly in terms of time spent when the objective function is more complex [34]. Although the RSA no longer tests all values within a parameter range, randomly selected sample points in the search range may ignore optimal values [35]. The BOA is one of the most popular methods for tuning hyperparameters in deep learning models [36]. Its main idea is that, given an objective function to be optimized, the posterior distribution of the objective function is updated by continuously adding sample points until the posterior distribution approximately corresponds to the true distribution or the function is executed for a predetermined number of iterations. It is a technique for adjusting hyperparameters based on the priori information, which is faster, more effective, and more efficient than the previous two algorithms. The major problem scenarios of the BOA are as follows:

X* = arg*_x_*_∈*S*_ max*f*(*x*) (3)

Here, S is the candidate set of x and f(x) is the objective function. The target of the BOA is to pick an x from S such that the value of f(x) is maximized or minimized.

The BOA was used to optimize the hyperparameters of the back propagation neural network (BPNN), AlexNet, VGG16, ResNet34, and IRBOA models. The activation function adopted for each model was ReLU with each batch_size set to 64, and the training epoch for the BPNN and CNN models were 5000 and 100, respectively. The cross-entropy function was employed for the loss function and the accuracy of the validation set was selected for the objective function of the BOA. The optimized variables are those proposed in 2.5.2, 2.5.3, and 2.5.4, including the number of neurons in the hidden layer of the BPNN (hidden), optimizer, learning_rate, the update interval in the learning rate decay algorithm (step_size), the multiplication factor for updating the learning rate (gamma), and L2 regular term parameters (weight_decay). Table 2 shows the search space of each hyperparameter.

#### 2.5.2. Optimizer

The optimizer is designed to minimize the loss in the training process through gradient descent, thereby enhancing the accuracy of the model. The stochastic gradient descent (SGD) algorithm and the adaptive momentum estimation (Adam) algorithm are two superior optimizers for image classification tasks in deep learning. Each of them has its advantages and disadvantages, hence the optimizer was selected to make the model optimal by employing the BOA in Section 2.5.1.

#### 2.5.3. Learning Rate

Learning rate is a very crucial hyperparameter in CNN classification models and impacts the recognition accuracy of the model. It is difficult and extremely important to choose the appropriate learning rate. In this paper, the model was trained by the equal-interval learning rate decay method, where the values of step_size and gamma were determined by BOA. The equation for the equal-interval learning rate decay is as follows.

(4) $$new\_lr=initial\_lr\times gamma^{\frac{epoch}{step\_size}}$$

where new_lr is the learning rate after decay, initial_lr is the learning rate before decay, gamma is the decay rate less than 1, epoch is the number of training rounds, and step_size is the decay step.

#### 2.5.4. Regularization

Regularization is performed by adding penalty terms for the loss function to reduce model complexity and instability to avoid overfitting the model. L2 regularization not only prevents overfitting but also makes the process of optimizing the solution stable and fast through weight decay. Therefore, the L2 regularization method was adopted to solve the problem of model overfitting, and the regular term parameter was calculated by BOA.

### 2.6. Performance Evaluation Indicators for the Model

Confusion matrix, accuracy, precision, recall, and F1-score are usually used to evaluate the performance of models for single-label image classification issues [37]. The confusion matrix is mainly used to compare the objective results with the predicted results when evaluating the recognition accuracy of the images. Accuracy refers to the probability of predicting correct samples among all samples. Precision indicates the proportion of samples with positive predictions that are correctly predicted. Recall denotes the proportion of correctly predicted outcomes in the actual sample of true examples. In the actual situation, precision and recall are mutually “restricted”. Therefore, we need the F1-score, a weighted average of precision and recall, to comprehensively evaluation the performance of models. The higher the F1-score, the better the performance of the model. The calculation formula of each indicator is as follows.

(5) $$\mathrm{Precision}\;(P)=\frac{TP}{TP+FP}$$

(6) $$\mathrm{Recall}\;(R)=\frac{TP}{TP+FN}$$

(7) $$\mathrm{Accuracy}\;(\mathrm{Acc})=\frac{TP+TN}{TP+TN+FP+FN}$$

(8) $$F1\text{-}\mathrm{score}=\frac{2\times P\times R}{P+R}$$

Here, TP is the number of samples where the actual case is true, and the predicted outcome is positive. TN is the number of samples where the actual case is true and the predicted outcome is negative, and the same for FP and FN. They can be calculated by a confusion matrix.

### 2.7. Experimental Environment

All models used in this study were trained and tested based on the Windows 10 operating system and the following specifications: Intel ^®^ Core™ i7-11800H CPU @ 2.30 GHz, 16 GB RAM, NVIDIA GeForce RTX 3060 GPU under CUDA v11.1 and cuDNN v8.0.5, PyTorch v1.9.0 (Facebook, America).

## 3. Results and Discussion

**Figure 6:**
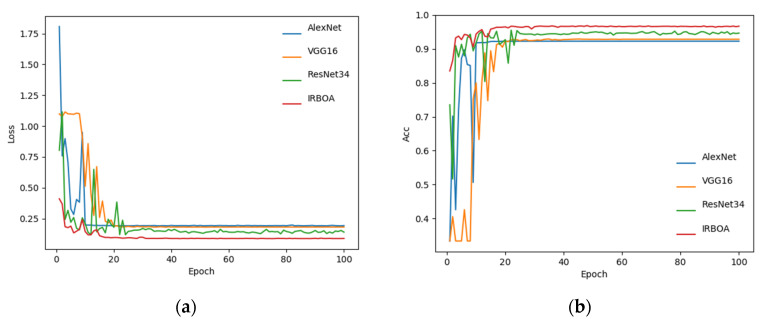
Comparison of the learning curves of the four CNN models. (**a**) Loss curve. (**b**) Accuracy curve.

Rice image datasets with different DOMs were trained on BPNN, AlexNet, VGG16, ResNet34, and IRBOA models. In addition, we compared the five models to find the optimal rice DOM inspection model. The training epochs for the BPNN and CNN models were 5000 and 100, respectively. Figure 6 shows the loss and accuracy curves of the four CNN models on the training set. The horizontal axis in the graph is the number of training epochs, and the vertical axes are the loss value (Loss) and accuracy (Acc) of the model, respectively. With the continuous increase of training epochs, the classification error of the training set shows a downward trend, and the accuracy shows an opposite trend. When the training epochs of the IRBOA model reach 69, the training loss is close to a stable value. The stable value of the average loss is 0.087, which is lower than the other three CNN models, and the accuracy is significantly higher than other models. In conclusion, the IRBOA model designed in this paper is reasonable and provides satisfactory training results.

**Figure 7:**
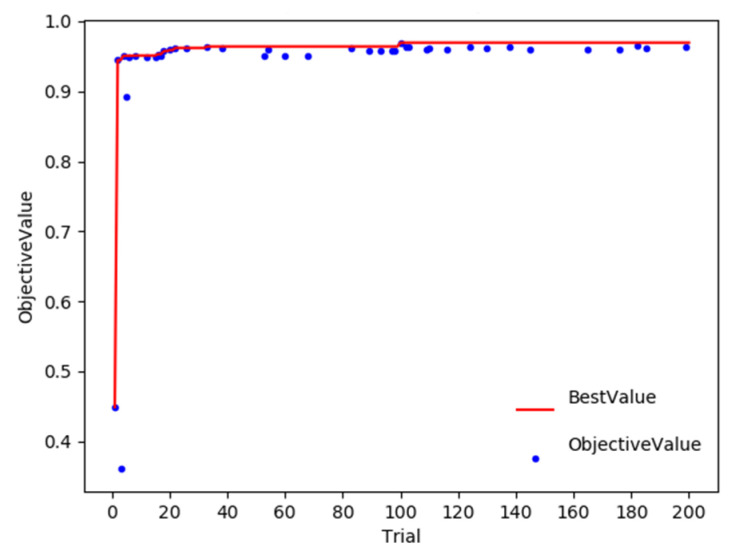
The Bayesian optimization process for the IRBOA model.

The hyperparameter optimization result of the IRBOA model is shown in Figure 7. The horizontal axis (Trial) in Figure 7 represents the number of iterations of the BOA, when it is 98, the objective function value is 0.9690 and the best result is obtained. However, the value of the objective function is still changing as the number of iterations increases. The effect indicates that the BOA is still trying to explore other optimal positions while approaching the optimal value. Table 3 lists hyperparameters obtained by the BOA for the five models, from which we can see that the hyperparameters are those that would normally not be set manually. The algorithm saves time and achieves results that cannot be captured by manual search. The models were trained and tested based on the optimized hyperparameters and the recognition rates were calculated for each model based on the test set. According to the comparative analysis in Table 3, we found that the detection accuracy of the IRBOA model for recognizing rice images was higher than that of the other four models, at 96.90%.

**Figure 8:**
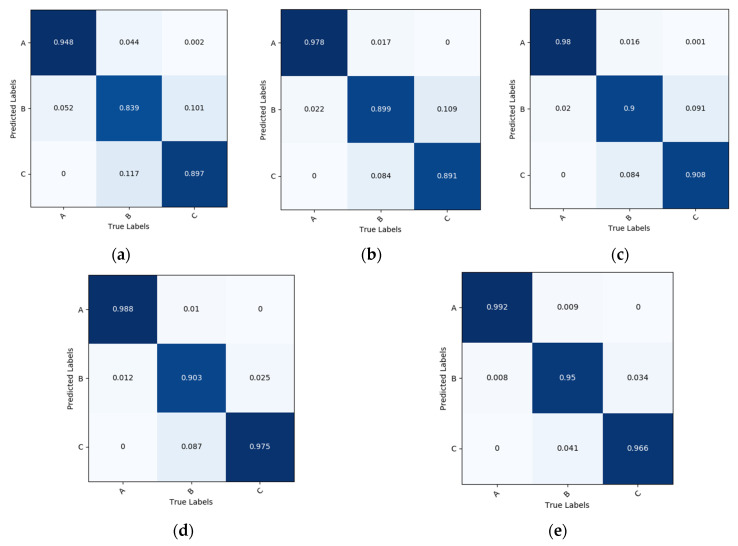
Confusion matrix of five models. (**a**) BPNN. (**b**) AlexNet. (**c**) VGG16. (**d**) ResNet34. (**e**) IRBOA.

Accuracy is not sufficient to describe the practical application performance of the model in the case of significant differences and imbalances in the data samples. Confusion matrices were plotted for several models based on the test set (Figure 8) to accurately assess the classification performance of the above five classification models for rice DOM. The actual categories (horizontal axis) are compared with the predicted category (vertical axis) in Figure 8 to depict the individual classification performance of each category. ‘A’ in the diagram for well-milled, ‘B’ for reasonably well-milled, and ‘C’ for substandard. These results demonstrated that the classification effect of the CNN models was better than that of BPNN, with the IRBOA model offering the best classification efficiency. The recognition precision of this model was 99.22%, 94.92%, and 96.55% for well-milled, reasonably well-milled and substandard rice, respectively, with an average correct detection rate of 96.90%. The accuracy of the IRBOA model is 7.41% higher than that of traditional machine learning and no less than 1.35% higher than that of the classic CNN models.

According to the prediction value in the confusion matrix, four different statistical indicators were attained, namely, TP, TF, FP, and FN. Moreover, the four evaluation indicators of accuracy, precision, recall, and F1-score, as well as the training time and single image test time of each model were calculated to compare the performance of several classification models (Table 4). The precision, recall, and F1-score of the IRBOA model were all 96.90% from Table 4. The corresponding values of BPNN, AlexNet, VGG16, and ResNet34 were all lower than the model proposed. Their F1-scores were 89.43%, 92.32%, 92.94%, and 95.59%. The experiments indicated that the recognition performance of the IRBOA model is better than that of the remaining four models, with higher accuracy and generalization performance. Meanwhile, we found that the BPNN took a longer time when testing the network on a single piece of data although its training time of it was much faster than the CNN model. The reason for this consequence is the BPNN takes a large amount of time in extracting the color and texture feature parameters and in reducing the dimension of the feature parameters using principal component analysis. The IRBOA model for recognizing rice DOM is characterized by its long training time, but high detection accuracy and less than 20 milliseconds for a single image among the four CNN models. The effect of the model proposed can meet the actual needs in terms of temporal and model recognition performance.

## 4. Conclusions

The nutritional value of rice decreases with the fineness of the rice DOM, while the processing process causes unnecessary food waste and affects national food security.

The purpose of this study was to solve the problems of the high labor intensity of traditional manual detection of rice DOM with manual feature extraction and a low recognition rate of existing classification methods based on machine learning. This paper presents an IRBOA model capable of extracting multi-scale rice features to identify classified rice DOM to further guide the processing process of rice enterprises.

The classical CNN model was improved by fusing the Inception-v3 structure and the residual structure. IRBOA, a multi-scale information fusion model, was constructed and its identification accuracy was enhanced relative to other classical networks. In addition, we used the BOA to seek the hyperparameters that led to the optimal performance of the model and increased the correct classification rate of the model. The IRBOA model, which performed hyperparameter optimization by BOA, achieved a recognition rate of 96.90% for rice DOM, while the testing time for a single image was less than 20 ms. The accuracy of IRBOA improved by 7.41 and no less than 1.35 percentage points relative to traditional machine learning methods and classic CNN models, respectively. The model enhances the feature representation and has better classification performance and generalization ability.

This study has demonstrated the feasibility of the inspection method proposed, which can provide a certain guidance to the processing work of rice enterprises and provide a reliable and accurate technical means for the classification of rice DOM level. More importantly, real-time rice DOM level evaluation can be achieved in the actual production process. Subsequently, the model can be combined with specific sorting apparatus to sort rice that has reached a certain DOM level in the rice milling section. It avoids the rice being over-milled in the next milling stage, so as to reach the goal of moderate processing and grain saving.

However, there are still some shortcomings in the research of this paper, and we will improve our current work in the following two aspects in the future work: (1) The model is prone to error attributed to the acquisition of single-sided images due to the different bran degrees on two sides of different DOMs rice. In the future, we will adopt the method of double-sided image acquisition [38] to improve the recognition rate of the model. (2) The chalky region of rice will have an impact on the discrimination of DOM level. In future research, we will search for effective image processing means to reduce the influence of the chalky areas of rice. (3) The accuracy of the model proposed only reaches 96.90%, which not only takes a long training time but also requires a large number of training samples. In the future, we can try to use the lightweight model [39,40] with small samples to save training time, or use the transfer learning model [41,42] to improve the recognition accuracy while reducing training time and samples.

## Figures and Tables

**Table 1:** Parameters of the IRBOA model structure.

Name of Layer		Parameters (Kernel_Size, Kernel_Num, Padding, Stride)
Image input		224 × 224 × 3
Inception-A	branch1	1 × 1 Conv, 8, 0, 1
	branch2	1 × 1 Conv, 12, 0, 1
		3 × 3 Conv, 24, 1, 1
	branch3	3 × 3 Maxpool, –, 1, 1
		1 × 1 Conv, 8, 1, 1
	branch4	1 × 1 Conv, 12, 0, 1
		3 × 3 Conv, 24, 1, 1
		3 × 3 Conv, 24, 1, 1
Filter concatenation		224 × 224 × 64
MaxPool		3 × 3 MaxPool, –, 1, 2
Residual-A1		–, 64, 1, 1
Residual-A2		–, 64, 1, 1
Residual-B1		–, 128, 1, 2
Residual-A3		–, 128, 1, 1
Residual-B2		–, 256, 1, 2
Residual-A4		–, 256, 1, 1
Residual-A5		–, 256, 1, 1
Inception-B	branch1	1 × 1 Conv, 64, 0, 1
	branch2	3 × 3 MaxPool, –, 1, 1
		1 × 1 Conv, 128, 0, 1
	branch3	1 × 1 Conv, 64, 0, 1
		1 × 7 Conv, 64, [0, 3], 1
		7 × 1 Conv, 128, [3, 0], 1
	branch4	1 × 1 Conv, 192, 0, 1
		1 × 7 Conv, 192, [0, 3], 1
		7 × 1 Conv, 192, [3, 0], 1
		1 × 7 Conv, 192, [0, 3], 1
		7 × 1 Conv, 128, [3, 0], 1
Filter concatenation		28 × 28 × 512
Avg_pool		1 × 1 × 512
Fc		3

“–” represents that there is no corresponding parameter.

**Table 2:** Hyperparameters search space based on BOA.

Model	Hyperparameter	Search Space
BPNN	hidden	{10, 12, 14, 16, 18, 20, 22, 24}
	optimizer	{SGD, Adam}
	learning_rate	[0.1, 0.00001]
	step_size	{600, 800, 1000, 1200, 1400}
	gamma	[0.1, 0.00001]
AlexNet, VGG16, ResNet34, IRBOA	optimizer	{SGD, Adam}
	learning_rate	[0.1, 0.00001]
	step_size	{10, 15, 20, 25, 30}
	gamma	[0.1, 0.00001]
	weight_decay	[0.1, 0.00001]

**Table 3:** Hyperparameter results for the five models of Bayesian optimization.

Model	Parameter (Hidden, Optimizer, Learning_Rate, Weight_Decay, Step_Size, Gamma)	Accuracy (%)
BPNN	12, Adam, 0.054, –, 1000, 0.00034	89.49
AlexNet	–, SGD, 0.035, 0.0001, 10, 0.0005	92.30
VGG16	–, SGD, 0.016, 0.00027, 20, 0.053	92.93
ResNet34	–, Adam, 0.00011, 0.00012, 25, 0.0001	95.55
**IRBOA**	**–, Adam, 0.00019, 0.0002, 15, 0.085**	**96.90**

“–” represents that there is no corresponding parameter.

**Table 4:** Detection performance indicators for the five models.

Model	Accuracy (%)	Precision (%)	Recall (%)	F1-Score (%)	Training Time (h)	Single Image Detection Time (s)
BPNN	89.49	89.44	89.43	89.43	0.10	20.69
AlexNet	92.30	92.35	92.30	92.32	0.56	2.87
VGG16	92.93	93.00	92.93	92.94	3.52	6.61
ResNet34	95.55	95.64	95.54	95.59	1.78	3.74
**IRBOA**	**96.90**	**96.90**	**96.89**	**96.90**	**4.22**	**12.93**

## Data Availability

The data presented in this study are available on request from the corresponding author.
